# A global and interoperable dataset of linguistic distributions derived from the *Atlas of the World’s Languages*

**DOI:** 10.1038/s41597-025-05828-6

**Published:** 2025-08-22

**Authors:** Peter Ranacher, Robert Forkel, Nour Efrat-Kowalsky, Matthias Urban, Antonia Hehli, Micha Franz, Gregory Biland, Aaron Kreienbühl, Alba Hermida Rodríguez, Matheus Azevedo, Martijn Romar, Andrea Klaussova, Takuya Takahashi, Nico Neureiter, Rik van Gijn, Meeli Roose, Outi Vesakoski, Robert Weibel, Gereon Kaiping, Sietze Norder

**Affiliations:** 1 University Research Priority Program (URPP) ‘Language and Space’, University of Zurich, Zurich, Switzerland; 2 Department of Geography, University of Zurich, Zurich, Switzerland; 3 Max Planck Institute for Evolutionary Anthropology, Department of Linguistic and Cultural Evolution, Leipzig, 04103 Germany; 4 Laboratoire “Dynamique du Langage”, UMR 5596, CNRS & Université Lumière Lyon 2, Lyon, France; 5 Ghent University, Ghent, Belgium; 6 Radboud University Nijmegen, Nijmegen, The Netherlands; 7 Leiden University Centre for Linguistics, Leiden, The Netherlands; 8 Copernicus Institute of Sustainable Development, Faculty of Geosciences, Utrecht University, Utrecht, The Netherlands; 9 Department of Geography and Geology, University of Turku, Turku, Finland; 10 School of Languages and Translation Studies, University of Turku, Turku, Finland

**Keywords:** Geography, Culture

## Abstract

Asher and Moseley’s *Atlas of the World’s Languages* illustrates the past and present spatial distribution of human languages across more than 100 maps. While the *Atlas* is an impressive resource, its data are not readily accessible for research. Language areas are presented as printed maps and referenced by name, rather than as digital spatial objects linked to a standardised language catalogue. To address these limitations, we present a digital dataset derived from the *Atlas*. We georeferenced the map images, digitised the language polygons in a Geographic Information System (GIS), and linked each polygon to a Glottocode — a unique identifier for languages and language varieties. Following the FAIR principles, we provide the data as a faithful digital replication of the Atlas (comprising 6,992 distinct language areas) and in enriched, aggregated versions for contemporary and traditional languages. The datasets capture the spatial distribution of human languages as depicted in the *Atlas*, with each polygon linked to an unambiguous identifier, enabling computational analyses of the origins, distribution, and drivers of global linguistic diversity.

## Background & Summary

The impressive *Atlas of the World’s Languages*^1^, henceforth *the Atlas*, depicts the spatial locations of over 6,000 languages and dialects across 108 maps, offering a comprehensive view of the geographic distribution of human languages. The *Atlas* illustrates contemporary linguistic diversity, broken down into ten geographic regions: North America, Meso-America, South America, Australasia and the Pacific, East and Southeast Asia, Southern Asia, Northern Asia and Eastern Europe, Western Europe, the Middle East and North Africa, and Sub-Saharan Africa. For North America, Meso-America, South America, and parts of Australasia and the Pacific, it also includes maps showing the geographic distribution of languages at the time of European contact, documenting the loss of diversity that set in after European colonization, and revealing the dynamics of language evolution across space and time.

While the *Atlas* provides immense cartographic and scientific value, it is not easily accessible for linguistic or geographic analysis, as all spatial information is encoded in printed maps. In contrast, geographic databases represent language areas—the locations where particular languages are spoken—as digital spatial objects that can be readily displayed on interactive maps, queried, and overlaid with other geographic information.

Considerable research effort has been devoted to cataloguing the diversity of human language and providing open language data in digital formats. Glottolog^2^ is a digital catalogue of the world’s languages, language families, and dialects, using Glottocodes as unique identifiers^3^. It has become the *de facto* standard for identifying languages, particularly those with limited documentation. Inventories such as WALS^4^, Grambank^5^, and PHOIBLE^6^ offer digital language data on grammatical and phonological features for many of the world’s languages. Similarly, efforts have been made in dialectometry to study the geographic distribution of words, grammar and sounds in regional language varieties^7^ and in toponomastics to document the origins and variation of place names^8^, revealing patterns linked to historical settlement, migration, and linguistic and cultural identity. Lastly, initiatives like the CLDF^9^ provide guidelines to make these data interoperable, while software packages like glottospace^10^ provide workflows for accessing, analysing and visualising these data.

Comparable attention has not been given to language maps, which often still exist only on paper or in non-interoperable formats, such as digital maps lacking proper geographic referencing. The absence of openly accessible, fully interoperable geographic speaker areas with global coverage has limited studies of language diversity to rely primarily on language counts within a given area^11^. The spatial range of language distribution has largely remained inaccessible, except through indirect proxies^12,13^. Glottolog does provide geographic information, but it represents languages as digital point locations—single latitude and longitude coordinates—rather than as language areas with defined boundaries. For example, Glottolog marks the Basque language with a single point in southern France but does not map the area spanning both Spain and France where Basque is spoken. In contrast, Ethnologue^14^ offers language-area polygons through its World Language Mapping System, employing the ISO 639-3 standard for language identification, which it also maintains. However, Ethnologue has faced criticism for its lack of scientific rigour, with entries often lacking proper academic references and metadata^3,15^. Furthermore, Ethnologue’s World Language Mapping System is behind a paywall, which limits both data accessibility and reproducibility^16^. This stands in contrast with the Open Science principles upheld by Glottolog, which offers free access to its data under a Creative Commons license.

Several recently published datasets provide digital language maps for specific geographic regions and language families, including the Caucasus^17^, North America^18^, Australia^19^, the Pacific^20^, and the Uralic languages^21^. The latter two projects have set a benchmark for language mapping by offering proper geospatial data—polygons and point geometries tied to a geographic coordinate reference system, along with comprehensive linguistic metadata and source attribution. What is still missing is a free, open and interoperable dataset of language polygons with global coverage.

To address this gap, we georeferenced and digitised the information shown in the *Atlas of the World’s Languages*^1^. In addition to the ‘raw’ data — the language distributions and attributes as presented in the Atlas — we enriched the dataset by linking each language area to a unique Glottolog entry. We provide aggregated versions of the enriched dataset based on the classification used in the *Atlas* and in Glottolog.

## Methods

Our dataset represents the language areas depicted in the Atlas of the World’s Languages^1^ as digital spatial objects conforming to the Polygon or MultiPolygon specifications of ISO 19125^22^. We first georeferenced the map images using a geographic information system (GIS), and then digitised the language polygons by tracing their outlines on the maps. Each polygon was linked to a Glottolog entry, either by directly matching it to the corresponding Glottocode, or, when this was not possible due to unclear language-dialect distinctions, by tentatively associating it with a Glottolog subgroup. The *Atlas* occasionally assigns multiple language labels to a single polygon, which we resolved by assigning such areas to the smallest Glottolog subgroup that includes all the referenced languages. We then implemented an error correction workflow to future-proof the data set and reduce maintenance complexity. Finally, we prepared the data for distribution. The polygons were divided into two datasets representing contemporary and traditional periods, and aggregated at both the language and language family levels. The final data were distributed as GeoJSON files and integrated into a CLDF package.

### Georeferencing the map images

We extracted all language map images from the source publication and scanned them at a resolution of 400 DPI to ensure sufficient detail. The scanned images were saved as TIFF files, using the map name in the figure caption as the filename. Next, we georeferenced the map images. Georeferencing involves assigning geographic coordinates to the map image, allowing it to be accurately positioned in a Geographic Information System (GIS). We performed georeferencing using the Georeferencer plugin in QGIS. We opened the map image alongside a base map — a reference map depicting landforms and administrative boundaries. Distinctive features, such as coastal turns or river estuaries, clearly visible in both the language map image and the base map, were used to place control points. We distributed the control points evenly across the image until the distinctive features in both maps were accurately aligned. If noticeable distortions appeared in certain regions, we added additional control points to those regions. Given the unknown projection of the map images, we used the Thin Plate Spline (TPS) interpolation method^23^, which applies piecewise polynomial functions to fit the map to the control points, allowing for localised deformations. We exported the georeferenced language maps as a GeoTIFF in the Spherical/Web Mercator projection (EPSG:3857)^24^, which is commonly used for displaying maps on a global scale.

### Digitising the language polygons

**Fig. 1:**
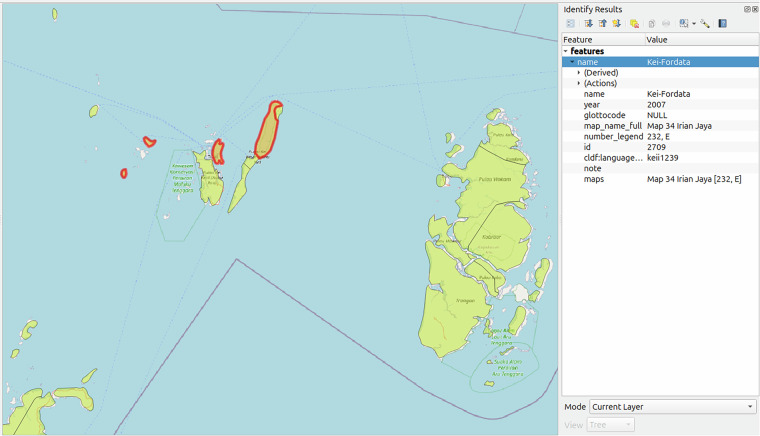
Digitised language areas from the *Atlas* overlaid on an OpenStreetmap base layer in QGIS. The language *Kei-Fordata* (Glottolog’s *Kei*) is spoken in four disjoint areas (outlined in red), represented by a single GIS object with a *MultiPolygon* geometry. The metadata is displayed in the panel on the right.

Digitising involves tracing the outlines of language areas on the georeferenced map images and converting them to valid Polygon and MultiPolygon geometries^22^ (see Fig. 1). We focused on languages depicted as areas and omitted any languages represented as point locations, such as circled labels indicating the approximate locations of small language enclaves. We digitised the language areas in QGIS using the Advanced Digitising toolbar. Rather than manually tracing the polygons from scratch, we used the Split Features Tool with snapping disabled to cut them from Natural Earth’s Physical Vectors^25^. The Physical Vectors dataset consists of earth’s land and major island polygons, created at a resolution of 1: 10^7^. Cutting polygons from this dataset ensures that the language areas align accurately with landmasses and coastlines. If a language area was entirely contained within another, we used the Add Ring tool to create a hole and the Add Polygon Feature tool to fill it with a new polygon representing the contained language, this time with snapping enabled.

After digitising a polygon, we filled in the attribute information using the Edit Feature form. We recorded the map name, the language name as shown on the map, and the language’s number from the legend. We also recorded the reference year, which — for contemporary maps, and if not otherwise specified, was the publication date, while for historical maps, it referred to the date corresponding to the time period depicted (e.g., *time of contact*). Additionally, we added comments, such as when the area’s outline was unclear or poorly visible on the map. After digitising all areas on a map image, we aggregated polygons representing the same language into a *MultiPolygon* — a combined geometry consisting of multiple disjoint polygons. For example, Kei-Fordata in Fig. 1 is spoken in four disjoint areas across four islands off the coast of Irian Jaya, which we digitised individually and then merged into a MultiPolygon. We ran the Check Validity tool to identify any invalid geometries and repaired them using the Fix Geometries tool. Finally, we exported all polygons and MultiPolygons in GeoJSON format in WGS 84 coordinate reference system (EPSG:4326).

### Enriching Polygons with Glottolog Data

Identifying languages by name is often impractical and ambiguous, as evidenced by the efforts of language classification systems such as Ethnologue^14^, WALS^4^, ISO 639-3^26^, and more recently, Glottolog^2^. While this problem is somewhat mitigated in the *Atlas* through the inclusion of classificatory information for each language, ambiguous language identification remains one of the major obstacles to data reuse. In addition, genealogical classification is often uncertain and even more prone to change than the names used to refer to a language in the literature, particularly for the lesser-described languages of the world. Thus, linking the areas depicted in the *Atlas* to unambiguous identifiers for languages and language varieties is one of the major contributions of our dataset. Glottocodes are unique identifiers for “languoids” (Glottolog’s cover term for languages, dialects and language subgroups^3^) maintained by Glottolog. They provide a standardised and convenient way to reference specific languages and dialects. Using a large language model (LLM), web crawling, and spatial analysis, we assigned a corresponding Glottocode to each language polygon.

#### Direct matching to corresponding Glottocode

First, we identified candidate Glottocodes based on geographic proximity. We created a 1,000 km spatial buffer around each language polygon and selected all language- or dialect-level Glottocodes with geographic point locations - obtained from Glottolog - that fell within this buffer. Since the distinction between languages and dialects is often blurry, we added all parent and child languoids as classified in Glottolog of any of the candidates to the candidate list as well. This approach assumes that parent and child languages are spoken in similar geographic areas, which may not always be accurate. However, since the candidate Glottocodes were further filtered and the Glottocode matches were validated later, a moderate overshoot was deemed acceptable at this stage.

Next, we used an LLM to identify the most plausible match among the spatially proximate candidate Glottocodes. We used the Anthropic Python library^27^ to access the Anthropic REST API and its families of large language models. In each prompt to the API, we queried the Claude 3 Opus model to determine the most plausible Glottocode among the candidates.

We parsed the LLM’s response and performed an initial sanity check to ensure the Glottocode was plausible. This involved confirming that the LLM did not hallucinate by returning a Glottocode outside the candidate set and that the response was not empty. If the Glottocode passed the sanity checks, we proceeded with validation. If the Glottocode failed these checks, we resorted to a Wikipedia query. Specifically, we used the Python MediaWiki package^28^ to query the English Wikipedia for language entries by concatenating the language name with the word ‘language’ in the search string. We then considered the top result and parsed the corresponding Wikipedia page for Glottocode information. Glottocodes are listed in a special infobox on Wikipedia, which can be extracted programmatically. After retrieving the Glottocode, we verified whether it was among the spatially proximate candidates before proceeding with further validation.

For validation, we compared the language name on the map with any alternative names that the language listed under the identified Glottocode might have. Glottolog provides highly structured metadata for its entries, including a list of alternative names by which the language is known. We parsed the GitHub site associated with the matched Glottocode and verified that the language name on the map appeared among these alternative names. In some cases, we encountered minor spelling differences between the language name on the map and an alternative name. We manually matched these names where appropriate.

For all polygons where automatic querying did not yield a Glottocode — either because the LLM or Wikipedia query failed to return a Glottocode, or because validation was unsuccessful — we manually searched for the Glottocode. We considered alternative names from the literature, spatial cues, and the classification information available in the *Atlas*. Our searches included Glottolog, the scientific literature, and general Internet sources.

#### Assigning unmatched polygons to Glottolog subgroups

Finding matching Glottolog languoids as counterparts for languages in the *Atlas* failed for 5–10% of the polygons. This was often due to the blurry language-dialect distinction. For example, the *Atlas* might list dialects which are not (yet) included in Glottolog’s somewhat patchy dialect repertoire. In such cases, we used the classification information provided in the *Atlas* to link the polygon to a Glottolog subgroup, which could be inferred to contain the corresponding Atlas language. For example, *Zalage* is listed in the *Atlas* as a dialect of *Mpo* in the *Maka-Njem* group of the *Benue-Congo* family. We applied this information to link *Zalage* to Glottolog’s *Mpoic* subgroup, a subgroup of *Makaa-Njem (A.80)* that includes languages and dialects such as *Esel*, *Bekwil*, and others listed as sister varieties of *Zalaga* in the *Atlas*. Since we later aggregate the polygons at both the language and family levels, this approach ensures maximum completeness and coverage, avoiding gaps due to missing Glottolog links. When an area is known to be populated by speakers of a particular language family, we communicate this information to users so they are not left wondering whether the area is, in fact, uninhabited. Such coarse-grained information at the subgroup level is useful, for example, when studying similarities between languages, which might be attributed to common descent or borrowing between languages from different families.

#### Handling language areas assigned to multiple languages

The *Atlas* sometimes assigns multiple numeric language labels to the same polygon. This likely indicates uncertainty about the exact borders between language areas, or the perforated nature of these borders, rather than suggesting that all referenced languages are spoken throughout the entire area. The case of Australia at the time of contact, in particular, supports this interpretation: on Map 35, the labels are distributed across the polygon, assigning languages to approximate locations *within* its boundaries.

We addressed this issue by assigning such areas to the smallest Glottolog subgroup that encompasses all referenced languages or dialects, in line with the Atlas’s overall mapping philosophy. Most maps in the *Atlas* do not have overlapping polygons, even though, as the editors note, “there is almost always a degree of overlap between areas where a language or languages predominate”^1^. As a result, the depicted areas are systematically biased, appearing smaller than they actually are, as multilingual regions are not adequately accounted for. Assigning polygons with multiple labels to the smallest subgroup encompassing all referenced languages aligns with the Atlas’s tendency to err on the side of “too small” in terms of area representation. If we had instead assigned the entire area to each individual language, we would have introduced a different bias that inflates area sizes. We also retained the original labels and Glottocodes for each individual language ascribed to the polygon as metadata.

### Error Correction Workflow

During initial quality control, we identified three main types of errors that could be addressed straightforwardly in an error correction workflow: mistakes in copying the data, incorrect matching of polygons to Glottocodes, and errors inherent in the original dataset. We implemented a workflow to correct these issues, thereby future-proofing the dataset and reducing maintenance complexity.

#### Correcting mistakes in copying the data

We encountered two main sources of error when copying the data: (1) erroneous polygon merges due to incorrect label assignments and (2) incorrect handling of enclaves. We implemented the following correction workflows to address both issues, which can be used to release updated versions of the data.

(1) A single language may have several disjoint polygons on the same map. We merged these polygons to MultiPolygons based on their numeric map labels to reduce the number of individual shapes. If labels were incorrectly transcribed from the *Atlas*, the resulting merge was incorrect as well. For example, one polygon for the Australian language Bayali with label 394 was erroneously recorded as 349 and merged with the polygon with that label. To address this error, we maintain an extendable configuration file of polygons with incorrect labels, which need to be merged to a different shape. Each incorrectly merged polygon can be specified by its unique identifier and a geographic point coordinate within the polygon. The correct polygon it should be merged with can be specified via its identifier.

(2) The *Atlas* sometimes uses circled labels to indicate the approximate locations of small language enclaves, rather than delineating proper area boundaries. Such labels may be mistakenly assigned to the surrounding polygon. This error can be addressed by “subtracting” the shape of the incorrectly assigned polygon from the correctly assigned polygon with the same label. Again, we maintain a configuration file listing all such error cases ensuring that all changes made are traceable.

The code to implement these workflows and look up configured corrections during the aggregation process for the CLDF data is invoked from the CLDF creation pipeline, which is implemented using the cldfbench package^29^.

#### Correcting Glottocode assignments

Identifying the correct Glottocode was often ambiguous and error-prone and will likely need updates as new releases of the Glottolog catalogue become available. In response, we implemented a workflow that enables quick and easy correction of any errors in the assignment. We listed the metadata in a CSV table, with each row linked to a polygon geometry in the file via a unique identifier. When aggregating the unmodified data to polygons at the language and family levels, metadata were retrieved from the table and geometries from the GeoJSON, making it straightforward to update metadata for any given (multi-)polygon. Tables are human-readable and well-suited to version control with tools such as Git, which are additional advantages of this curation model.

#### Handling conflicting information and evident mistakes in the source

As a general rule, we stayed true to the source, but we deviated from this principle in the case of 1) conflicting information in maps covering the same region and 2) obvious mistakes.

1) Several maps in the *Atlas* often cover the same region, and occasionally, language areas are depicted differently in these overlapping maps. In such cases, we selected one map as the source, disregarding conflicting information from the others. For example, the region north-west of Lake Chad is shown in *Map 73 North-West Africa* and, with much higher resolution, in *Map 99 Lake Chad Region*. The choice of map was often a pragmatic one. While the more detailed Map 99 provides more language polygons, the languages it shows beyond those in Map 73 could not be reliably identified. Thus, we opted for the coarser areas depicted in Map 73.

2) Any work at the scale of the *Atlas* will inevitably contain mistakes. Using the error correction workflow described above, we have addressed and corrected obvious mistakes when identified. As outlined in the validation section, we do not automatically flag information that conflicts with other sources as errors. However, determining what constitutes an “obvious” mistake requires a judgement call. Clear examples of errors we corrected include language areas that share the same legend number but appear in different colours, such as the two polygons labelled 67 on *Map 10 Meso-America: Time of Contact*.

### Aggregation

We provide the data in two forms: (1) as unmodified (raw) polygons in GeoJSON format, faithfully copied from the Atlas, and (2) as polygons enriched with Glottocodes in CLDF format^9^. The enriched date are provided at three levels of aggregation: (a) as *Features*, speaker areas retaining the Atlas’s classification, (b) as *Language Areas* aggregated at the language level based on Glottolog’s classification, and (c) as *Language family areas* aggregated at the language family level based on Glottolog’s classification.

The *Atlas* often uses a different classification system than Glottolog: what the *Atlas* considers a language may be classified as a dialect in Glottolog, and vice versa. The three levels of aggregation account for this discrepancy. The unaggregated speaker areas (a) use Glottocodes to identify languages but otherwise strictly follow the Atlas’s classification. For example, they may include areas that Glottolog classifies as dialects, simply because the *Atlas* treats them as separate languages. In contrast, the aggregated speaker areas in (b) and (c) follow Glottolog’s classification, grouping all speaker areas below the language or family level accordingly.

All enriched and aggregated data are provided in CLDF format^9^, with the geodata serialised as GeoJSON files, and linked into the CLDF package via the MediaTable component. CLDF is particularly well-suited as a distribution format because it includes a LanguageTable component for listing all relevant languoids (i.e., languages, language groups, or dialects), supports unambiguous links to Glottolog, and allows speaker areas to be linked through the speakerArea component.

#### Contemporary and traditional language areas

As stated by the editors, the “principal concern of the atlas is the contemporary distribution of the world’s languages”^1^. However, they deemed the effects of the “colonization of distant parts of the world by maritime nations of Europe from the seventeenth century onwards” both disruptive and recent enough to complement the *Atlas* with maps of the Americas and Australia at “time of contact”, depicting the traditional distribution at the very start of colonization. For other parts of the world, no “time of contact” maps are provided.

In our dataset, we aim to provide comparable language areas with global coverage for both contemporary and traditional periods. For the Americas and Australia, the *Atlas* explicitly distinguishes between these. For the remaining regions – Eurasia, Africa, and the Pacific – we treat the areas as representing contemporary and traditional periods and include them in both language area sets while acknowledging but setting aside the potential disruptions caused by more recent historical events in these regions. Having comparable language area sets with global coverage for contemporary and traditional periods is advantageous and facilitates analyses in historical linguistics, language evolution, and diversity linguistics, especially since the disruption of societies caused by colonialism may violate assumptions of uniformitarianism^30^. For example, the data could help quantify the loss of linguistic diversity in terms of lost geographical area, rather than solely by the number of remaining speakers. They could also support testing hypotheses about geographical barriers and facilitators of language spread, or assist phylogeographic analyses by serving as areal priors when inferring the spatio-temporal diffusion of a language family.

## Data Records

**Fig. 2:**
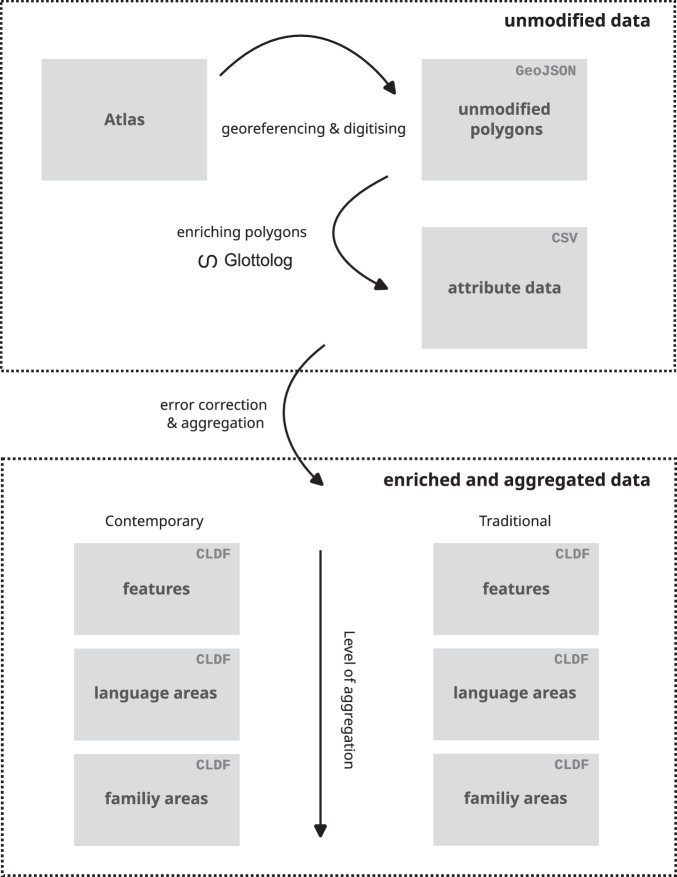
Data sets (grey rectangles) and methods used to derive them (black arrows). The unmodified raw data include digitised language polygons in GeoJSON format and attributes with links to Glottolog in CSV format. The enriched and aggregated data include contemporary and traditional language polygons enriched with Glottocodes at increasing levels of aggregation: features, language areas, and family areas.

The dataset^31^ derived from the *Atlas of the World’s Languages* is curated in a public repository on GitHub (https://github.com/glottography/asher2007world) as part of a larger dataset of language areas from other published sources. Released versions are archived with and published on Zenodo (10.5281/zenodo.15287258). We digitised 6992 distinct language areas from the *Atlas*. We provide the data in an unmodified raw format, as a faithful copy of the source data, and in an enriched and aggregated format based on the Glottolog language and family levels (see, Fig. 2).

### Unmodified data

The unmodified raw data includes the language areas and attributes faithfully copied from the source maps and serves as input to the error correction workflow described above, which is then followed by enrichment and aggregation. We provide the language areas as GIS vector objects in the GeoJSON format as file raw/dataset.geojson in the package deposited on Zenodo. Each language area is represented as a simple polygon geometry if it consists of a single contiguous area or as a MultiPolygon geometry if it includes multiple disjoint areas. When multiple maps show the same language, the corresponding areas are generally combined into a single MultiPolygon based on matching *name* attributes, provided they represent the same time period (contemporary vs. time of contact) and are not marked as multilingual. Multilingual areas are represented as duplicate shapes with distinct metadata.

The metadata associated with the shapes is available as a CSV table in etc/features.csv. The CSV contains attributes of the language polygons in the GeoJSON file, including the language name, the map name, and the year the map represents, and links them with Glottolog languoids by assigning a Glottocode. Any comments on this assignment can be added as notes. Table 1 provides a summary of all attributes included in the CSV file. If areas span across multiple maps, all map names and associated numeric language labels are listed, separated by a vertical bar. If specified in the map, the year is either set to *contemporary* or *time of contact*; if not specified, it is set to the year of publication, i.e., *2007*. The metadata in the CSV file serves as the authoritative source and is carried through during the enrichment and aggregation of the language polygons. It is easily editable, allowing the linguistic community to correct any metadata inaccurately copied from the *Atlas* or any misassigned Glottocodes. While the GeoJSON file also includes metadata – except for the Glottocodes – to support traceability of the aggregation process, it should not be edited, as any changes will be ignored during aggregation.

**Table 1:** Attributes provided in the CSV file.

Attribute	Description	Example
**id**	A unique identifier for each (multi)polygon.	2526
**name**	The name of the language as on the map.	Kanamaré
**number_legend**	The numeric language label assigned to the language.	121
**map_name_full**	The full name of the map.	Map 16 Western Brazil: Time of Contact
**year**	The year the language area refers to.	contemporary
**glottocode**	The Glottocode assigned to the language area.	yine1239
**note**	Comments on the Glottocode assignment.	An extinct Yine language.

### Enriched and aggregated data

We packaged the data in the Cross-Linguistic Data Format (CLDF), a standard format for historical and typological language data^9^. Since the *Atlas* includes two sets of maps, one showing historical speaker areas at the time of European contact, and the other showing contemporary areas, we partitioned the data into two separate datasets for distribution, one for traditional and one for contemporary language areas. Each CLDF dataset includes three sets of vector geometries enriched with Glottocodes at three levels of aggregation in GeoJSON format:

1. **Features**: speaker areas retaining the Atlas’s classification
2. **Language areas**: speaker areas aggregated at the language level based on Glottolog’s classification
3. **Family areas**: speaker areas aggregated at the language family level based on Glottolog’s classification

The set of contemporary speaker areas includes 5573 features, aggregated to 3999 language areas and 190 family areas. The set of traditional speaker areas includes 6095 distinct features, aggregated to 4382 language areas, and 228 family areas.

The metadata for the vector geometries are listed in a *ContributionTable*, including a link to each GeoJSON file. A *LanguageTable* enumerates the language- and family-level languoids represented in the dataset. These languoids are linked to features in the source, either directly mapped to the languoid or associated with parts of the languoid, such as dialects or subgroups.

### Interoperability

The primary value of the dataset lies in the comprehensiveness of the source data and the interoperability added through digitisation and language classification. The editors of the *Atlas* coordinated contributions from regional experts and integrated data from a wide range of sources, resulting in near-global coverage of language areas—though with varying levels of detail across regions. We further enriched the source data by linking the speaker areas depicted in the *Atlas* to Glottolog’s language catalogue and classification, which enables full interoperability. This, in turn, allows for seamless integration and comparison with other datasets. For example, one could easily swap the Austronesian language areas in the *Atlas* with the more detailed regions derived from Wurm & Hattori’s *Language Atlas of the Pacific Area*^20^, increasing the number of covered languages by roughly 50. Similarly, the relatively coarse areas assigned to Pama-Nyungan languoids could be replaced with the finer-grained delineations described in Bowern (2021)^32^, raising the number of distinct languages by about 100. Thus, the *Atlas* data serve as a backbone for programmatically integrating additional sources, enabling researchers to assemble a global speaker area dataset tailored to specific research problems.

The potential for interoperability already became evident during validation. We computed the overlap of areas matched to the same Glottocode in the *Atlas* and Wurm & Hattori’s *Language Atlas of the Pacific Area*, both of which are available as CLDF datasets. We found mismatches in the source data of both publications, which we addressed using the error correction workflow.

## Technical Validation

This dataset provides a digital and enhanced representation of the *Atlas of the World’s Languages*, linking its language areas to unique entries in the Glottolog language classification system. To establish the validity of the data for scientific analysis, we must 1) validate the unmodified data to ensure they faithfully represents the maps in the *Atlas*, 2) validate the syntactic correctness and referential integrity of the distributed data to ensure it is accessible computationally and 3) validate the Glottolog matching to ensure the data provide correct mappings to Glottolog languoids.

### Validation of the unmodified data

During data collection, detailed validation and quality checks were performed to ensure that the digitised speaker areas were faithful representations of the areas depicted in the *Atlas*, as well as valid GIS objects. Table 2 outlines potential issues encountered during data collection and automatic and manual validation to identify and resolve them.

**Table 2:** Potential issues during data collection and checks to resolve them.

Potential Issue	Automatic validation	Manual validation
The **geometry** of the language polygon was traced incorrectly.	Perform a geometry check and fix any invalid geometries.	Visually inspect the digitised geometry against the language area in the source map.
The language **name** was copied incorrectly.	Verify that a language with the name copied from the map exists on Glottolog.	Cross-check the copied name with the source map.
The numeric language **label** was copied incorrectly.	Verify that the copied numeric label exists in the map legend.	Check the copied label against the source map legend.

### Validation of the distributed data

The dataset is distributed in CLDF format, which provides the cldf validate command, run in the UNIX shell, for validating the syntactic correctness and referential integrity of the data. If completed successfully, cldf validate ensures that (1) the GIS objects formatted as GeoJSON can be read from the dataset, (2) the GIS objects can be associated with rows in the language table and (3) the Glottocodes provided in the language table are valid, well-formed and listed in the corresponding Glottolog release.

We also ran the cldfbench geojson.validate command from the cldfgeojson package to perform a final validation of the aggregated geometries for the language and family areas, after they were merged from (potentially) multiple features in the source publication.

### Validation of the Glottolog matching

To validate the matching of GIS objects to Glottolog languoids, we used two methods pioneered in^20^: (1) we checked the spatial distances between speaker areas as digitised from the *Atlas* and corresponding Glottolog point locations and (2) we checked the spatial extent of Multipolygons mapped to the same Glottocode.

1) We computed the pairwise spatial distances between the (Multi)Polygons aggregated at language level and Glottolog’s point locations for the corresponding language. A distance of zero indicates that the Glottolog location falls within the speaker area from the *Atlas*, thereby corroborating the Glottolog mapping. While a distance greater than zero does not necessarily imply an incorrect mapping, it hints at a potential discrepancy. We manually examined all cases where the distance exceeded two degrees or about 200 km near to the equator.

2) We computed the spatial extent of the constituent parts of Multipolygons mapped to the same Glottocode. MultiPolygons whose parts were scattered over a large area were further inspected. This approach could identify potential issues during digitisation, such as numeric language labels incorrectly copied from the map or incorrect Glottocodes assigned to a speaker area. It also helped identify errors in the *Atlas* itself – for example, cases where the legend label in the source was wrong. While we generally took the information in the *Atlas* as authoritative, we corrected these issues when found.

## Usage Notes

GeoJSON is a widely supported format for handling spatial data and can be easily accessed using GIS software or spatially enabled programming languages. In Python, the Geopandas package makes it straightforward to read these files directly into a GeoDataFrame using the read_file() function, from where the spatial data can be manipulated, analysed, and visualised. In R, the sf package supports reading and handling spatial formats. Its st_read() function imports GeoJSON files into R, enabling the spatial data to be seamlessly integrated into the tidyverse ecosystem for statistical manipulation and visualisation, using packages such as dplyr and ggplot2

QGIS, a comprehensive open-source Geographic Information System, provides a user-friendly interface for working with GeoJSON files. Users can drag and drop the files into the QGIS workspace or use the “Add Layer” feature to import them. Once loaded, QGIS offers extensive tools for visualising, analysing, and editing the spatial data.

Any errors identified during the reuse of this dataset are kindly requested to be submitted to https://github.com/glottography/asher2007world/issues to facilitate transparent corrections in future releases.

## Data Availability

The code used to generate the CLDF datasets from the unmodified data is included in the dataset’s repository (https://github.com/Glottography/asher2007world/blob/main/cldfbench_asher2007world.py) as part of the released version. It relies on the pyglottography package, which is available via the Python Package Index (PyPI) at https://pypi.org/project/pyglottography/. The experiments described in the Technical validation section make use of the validation methods provided by the cldfgeojson package, again available on PyPI https://pypi.org/project/cldfgeojson/.
